# Mechanism of dorsal root ganglion stimulation for pain relief in painful diabetic polyneuropathy is not dependent on GABA release in the dorsal horn of the spinal cord

**DOI:** 10.1111/cns.13192

**Published:** 2019-07-23

**Authors:** Eva Koetsier, Glenn Franken, Jacques Debets, Lonne Heijmans, Sander M.J. van Kuijk, Bengt Linderoth, Elbert A. Joosten, Paolo Maino

**Affiliations:** ^1^ Pain Management Center, Neurocenter of Southern Switzerland Regional Hospital of Lugano Lugano Switzerland; ^2^ Division of Anaesthesiology Department of Acute Medicine Regional Hospital of Lugano Lugano Switzerland; ^3^ Department of Anesthesiology and Pain Management Maastricht University Medical Center+ Maastricht The Netherlands; ^4^ Department of Translational Neuroscience, School of Mental Health and Neuroscience (MHeNS) University of Maastricht The Netherlands; ^5^ Muroidean Facility School of Cardiovascular Diseases (CARIM) Maastricht The Netherlands; ^6^ Department of Clinical Epidemiology and Medical Technology Assessment Maastricht University Medical Center+ Maastricht The Netherlands; ^7^ Department of Clinical Neuroscience Karolinska Institutet Stockholm Sweden

**Keywords:** animal model, dorsal horn, dorsal root ganglion stimulation, painful diabetic polyneuropathy, rats, spinal cord, γ‐aminobutyric acid

## Abstract

**Aims:**

It is hypothesized that dorsal root ganglion stimulation (DRGS), sharing some of the mechanisms of traditional spinal cord stimulation (SCS) of the dorsal columns, induces γ‐aminobutyric acid (GABA) release from interneurons in the spinal dorsal horn.

**Methods:**

We used quantitative immunohistochemical analysis in order to investigate the effect of DRGS on intensity of intracellular GABA‐staining levels in the L4‐L6 spinal dorsal horn of painful diabetic polyneuropathy (PDPN) animals. To establish the maximal pain relieving effect, we tested for mechanical hypersensitivity to von Frey filaments and animals received 30 minutes of DRGS at day 3 after implantation of the electrode. One day later, 4 Sham‐DRGS animals and four responders‐to‐DRGS received again 30 minutes of DRGS and were perfused at the peak of DRGS‐induced pain relief.

**Results:**

No significant difference in GABA‐immunoreactivity was observed between DRGS and Sham‐DRGS in lamina 1‐3 of the spinal levels L4‐6 neither ipsilaterally nor contralaterally.

**Conclusions:**

Dorsal root ganglion stimulation does not induce GABA release from the spinal dorsal horn cells, suggesting that the mechanisms underlying DRGS in pain relief are different from those of conventional SCS. The modulation of a GABA‐mediated “Gate Control” in the DRG itself, functioning as a prime Gate of nociception, is suggested and discussed.

## 1 INTRODUCTION

In the Western world, the prevalence of chronic pain is 30%,^1^ and about one‐fifth of these persons has predominantly neuropathic pain.^2^, ^3^, ^4^ Neuropathic pain is described as pain caused by a lesion or a disease of the somatosensory nervous system.^5^ One of the most common neuropathic pain conditions is diabetic polyneuropathy (DPN), which is most typically a chronic, symmetrical, length‐dependent sensory‐motor polyneuropathy.^6^ Up to 1/3 of all patients with diabetes acquire painful diabetic polyneuropathy (PDPN),^7^ and pharmacological pain therapy is often insufficient in these patients.^8^ Conventional spinal cord stimulation of the dorsal columns (hereafter labeled SCS) has resulted in significant pain reduction in many intractable PDPN patients.^9^, ^10^, ^11^ However, in about 40% of these patients SCS treatment is not effective.^9^, ^10^, ^11^ Conventional dorsal root ganglion stimulation (hereafter named DRGS) acts at the level of the dorsal root ganglion (DRG), a promising new location for neuromodulation in managing selected pain conditions—among those also PDPN.^12^ DRGS enables the physician to target a more peripheral, selective communication station for all nociceptive signaling from the peripheral nervous system to the dorsal horn in the spinal cord, from where the information is further processed via the spinothalamic tracts to the brain.^13^ The DRG plays an important role in both neuropathic and nociceptive pain conditions.^14^ Therefore, while SCS can theoretically only modulate Aβ fiber signaling, DRGS might also be able to modulate Aδ‐ and C‐type fiber signaling.^15^

A key molecule in the processing and modulation of the nociceptive signal in the spinal dorsal horn is γ‐aminobutyric acid (GABA).^16^, ^17^ Experimental data demonstrated decreased extracellular GABA levels and increased intracellular GABA levels in the dorsal horn after peripheral nerve injury.^18^, ^19^ GABA has been proposed to have a major role in nociceptive and non‐nociceptive processing according to the Gate Control Theory.^20^ Based on this, it was suggested to stimulate non‐nociceptive Aβ fibers in the dorsal columns (SCS) aiming to turn on the Gate mechanisms and modulate the nociceptive input. In an experimental study of allodynic rats, increased levels of extracellular GABA were indeed found in the dorsal horns in response to SCS*.* 18, 21, 22 The pivotal role of GABA in the analgesic effect of SCS has furthermore been confirmed by the fact that the application of the GABA_B_ agonist baclofen changed “non‐responders‐to‐SCS” into “responders‐to‐SCS.”^23^, ^24^, ^25^, ^26^ The results of an experimental study of Janssen et al^27^ demonstrated additionally that responders‐to‐SCS showed decreased levels of intracellular GABA‐immunoreactivity (GABA‐IR) in the spinal dorsal horn in comparison with non‐responders‐to‐SCS and Sham‐SCS animals. A relation between the release of intracellular accumulated GABA in the spinal cord dorsal horn, and the analgesic effect of SCS was therefore hypothesized.^27^, ^28^, ^29^, ^30^

The mechanisms underlying DRGS and its ensuing pain relief are as yet unknown, and it is likely that it shares some spinal and supraspinal mechanisms with SCS, dependent on Aβ fibers activated by both types of stimulation. The present study is therefore aimed to investigate the hypothesis that DRGS induces GABA release from spinal dorsal horn cells. We used quantitative immunohistochemical analysis in an animal model of PDPN to study the effect of one single DRGS paradigm on the levels of spinal dorsal horn intracellular GABA.

## 2 METHODS

### 2.1 Ethical statement

The protocols for this study were approved by the Animal Care Committee of the Maastricht University Medical Centre (DEC 2013‐079). The procedures were conducted in accordance with the guidelines of the European Directive for the Protection of Vertebrate Animals Used for Experimental and Other Scientific Purposes (86/609/EU).

### 2.2 Animals

The study was performed on 48 adult female Sprague Dawley rats, weighing 170‐230 g and 8 weeks old at the start of the experiments (Charles River, Maastricht, The Netherlands).They were preoperatively housed in pairs and postoperatively individually, in transparent plastic cages situated in a climate controlled room under a 12‐hour light/dark cycle with food and water ad libitum. All efforts were made to minimize the number of animals used and their suffering, and alternatives to in vivo techniques were considered.

### 2.3 Induction of diabetes mellitus

All animals fasted overnight before the induction of diabetes. Streptozotocin (STZ, Sigma‐Aldrich, Schnelldorf, Germany) was freshly dissolved in sterile NaCl 0.9% to a solution of 65 mg/mL. Animals were intraperitoneally injected with the STZ solution (65 mg/kg) to induce diabetes mellitus (DM). On day 4 post‐STZ injection, blood glucose levels were measured from the saphenous vein, using an Accu‐Chek Aviva® glucometer (Roche Diagnostics GmbH, Mannheim, Germany). Rats developing diabetes, defined as a blood glucose level of ≥15 mmol/L,^31^ were included in the study.

### 2.4 Development and assessment of mechanical hypersensitivity

Pain behavior was assessed by testing mechanical hypersensitivity based on the hind limb paw withdrawal response to Von Frey filaments (bending forces 0.6, 1.2, 2.0, 3.6, 5.5, 8.5, 15.1, and 28.84 g). Rats were placed in individual cages with a wire mesh floor and were allowed to acclimate to the experimental set‐up for 15 minutes. The 50% withdrawal threshold (WT) was determined by use of the up‐down method,^32^ as previously described.^33^ The cutoff value was defined as the absence of the paw withdrawal response to a 28.84 g force to prevent tissue damage. The registered 50% WT’s (measured in grams) were then multiplied by 10 000 and logarithmically transformed to conform with Weber's law^34^ and in order to obtain a linear scale.

Mechanical hypersensitivity was tested at baseline (pre‐STZ injection) and weekly during 4 weeks post‐STZ injection. Mechanical hypersensitivity, caused by PDPN, was defined to be present in case of a decrease in ≥0.2 unit of the log^10^ (10 000 × 50% WT) when compared to baseline. Only animals that developed mechanical hypersensitivity were selected for this study and treated with DRGS.

### 2.5 Implantation of DRGS electrode

Under general anesthesia, bipolar electrode was implanted unilaterally at the L5 DRG, according to the earlier described procedure adapted from Pan et al.^35^, ^36^ In short, via a paravertebral incision the intervertebral foramen was exposed, the foramen was opened, and the anode and cathode were implanted at the DRG. The electrode was then secured into the transverse process followed by closure of the incision in layers.

### 2.6 DRGS

For comparison with previous experimental studies regarding neurostimulation in PDPN animals, we used time and design as in previous (published) experiments.^33^, ^36^ Animals were stimulated for 30 minutes at days 3 and 4 following implantation. The stimulation on day 3 was meant to establish the maximal pain relieving effect of DRGS,^36^ while the stimulation on day 4 was performed to activate the mechanisms of action before perfusing the animals at the “peak of DRGS‐induced pain relief.” For stimulation, the implanted electrode was connected to the pulse generator (A‐M systems MultiStim Model 3800, fitted with an A‐M systems 3820 stimulus isolator [A‐M systems, Sequim, WA, USA]). To assess motor thresholds (MT), the current amplitude was increased during stimulation at 2 Hz with a pulse width of 0.2 ms, defining MT as the current at which any further increase resulted in hind limb movement. Stimulation was delivered at a current set at 66.7% of MT (around 0.18 mA), a frequency of 50 Hz, and a pulse width of 0.2 ms. Amplitude was set at zero for sham stimulation.

On the third day following implantation, mechanical hypersensitivity was assessed pre‐DRGS, during stimulation at *t* = 15 minutes and *t* = 30 minutes, and 30 minutes post‐DRGS (*t* = 60 minutes). On the 4th day after implantation, rats received again 30 minutes of DRGS, just before being perfused (at the peak of the DRGS‐induced pain relief). An animal was defined as a “responder‐to‐DRGS” if it developed an increase in the log^10^ (10 000 × 50% WT) of ≥0.2 when compared to the pre‐DRGS baseline.^36^

### 2.7 Tissue preparation

At the peak of DRGS‐induced pain relief (after 30 minutes of stimulation), 4 responder‐to‐DRGS animals and 4 Sham‐DRGS animals were anesthetized with pentobarbital (100 mg/kg) and perfused transcardially with 15% picric acid and 4% paraformaldehyde in 0.2 M phosphate buffer saline (PBS; pH 7.6). A laminectomy was performed to extract spinal cord L4‐L6 regions, which were postfixated overnight (at 4°C) and cryoprotected in 10% sucrose for 24 hour, and then incubated for 72 hour in 25% sucrose in 0.1 M PBS (pH 7.6, at 4°C). Thereafter, tissues were frozen using solid carbon dioxide. Transverse cryosections (30 µm thick) were mounted on gelatine‐coated glass slides and stored at −20°C until staining. The cryosectioning was performed in a blinded fashion for treatment.

### 2.8 Immunohistochemical detection of GABA

Slides were allowed to dry at room temperature for 2 hour before being washed with Tris‐buffered saline (TBS, 0.1 M, pH 7.6), including 0.3% Triton X‐100 (TBS‐T), TBS, and TBS‐T. Thereafter, the slides were blocked for anti‐GABA immunohistochemistry by the incubation in 2% normal donkey serum (Sigma‐Aldrich, Zwijndrecht, The Netherlands, D9663) for 1 hour, diluted in TBS‐T, and then incubated with rabbit anti‐GABA polyclonal antibody (1:5000 diluted in TBS‐T; Sigma‐ Aldrich, Zwijndrecht, The Netherlands, A2052) for 48 hour. After rinsing unbound primary antibody with TBS, sections were incubated for 2 hour with the secondary antibody Alexa fluor 488 donkey anti‐rabbit IgG (1:100 diluted in TBS‐T; Invitrogen, Breda, The Netherlands, A21206). Lastly, slides were rinsed with TBS and coverslipped with TBS/glycerol (20%/80%).

### 2.9 Immunoreactivity analysis

After the GABA‐staining protocol was completed, sections were observed under an Olympus AX‐70 microscope and immunohistochemical analysis was performed, as previously described by Janssen et al.^27^ Firstly, photomicrographs were taken from both ipsilateral and contralateral dorsal horns for the spinal levels L4‐L6 using the Provis AX70 fluorescent microscope (Olympus) with a U‐CMAD‐2 black and white camera (Olympus) with CellP imaging software. Images were merged using Adobe Photoshop, and grayscale values were analyzed (blinded for treatment) using AnalySIS software. Regions of interest for the analysis of GABA‐IR were lamina of Rexed 1‐3 of the dorsal horn.^37^ Grayscale values were calculated for these laminae. The outcomes should depict the intracellular GABA content in the dorsal horns.

### 2.10 Statistical analysis

The assay and the data analysis were performed in a blinded fashion in an identical mode as published before.^27^ Data are represented as mean ± standard error of the mean (SEM). Statistical analysis of grayscale values was performed using GraphPad Prism software. A Wilcoxon signed‐rank test was used for comparison of mechanical hypersensitivity pre‐STZ injection and pre‐implantation. For comparisons of grayscale values between levels (L4 vs. L5 vs. L6), treatments (DRGS vs. Sham‐DRGS), and left‐right differences (ipsilateral vs. contralateral), a two‐way analysis of variance (ANOVA) was used, followed by a Sidak's multiple comparisons test. A *P* value < 0.05 was considered significant.

## 3 RESULTS

### 3.1 Description of cohorts of animals

Forty‐three of the 48 animals (90%) that were injected with STZ developed DM (blood glucose ≥15 mmol/L). Twenty‐two out of the 43 diabetic animals developed painful neuropathy (51%, ≥0.2 decrease in log^10^ (10 000 × 50% WT) and were implanted with a DRGS system. Two animals were excluded from the study because of high MT (MT > 1mA) and two due to a connector breakage. Eleven animals were selected for treatment with DRGS and seven for treatment with Sham‐DRGS.^36^ Of these animals, 4 responders‐to‐DRGS animals and 4 Sham‐DRGS animals were selected for the GABA‐IR analyses.

### 3.2 Development of PDPN and effect of DRGS on PDPN

In the DRGS group, the log^10^ (10 000 × 50% WT) dropped significantly from 5.059 pre‐STZ injection to 4.376 pre‐DRGS (*P* < 0.01). In the Sham‐DRGS group, the log^10^ (10 000 × 50% WT) dropped also significantly from 5.041 pre‐STZ injection to 4.416 pre‐Sham‐DRGS (*P* < 0.05).^36^ The mean log^10^ (10 000 × 50% WT) value of the animals that were selected for GABA‐IR analyses (*n* = 8) decreased from 4.99 ± 0.05 pre‐STZ injection to 4.50 ± 0.06 pre‐implantation (4 weeks post‐STZ injection, *P* < 0.01). Motor thresholds were tested prior to DRGS (to select the appropriate amplitude). Normal responses were observed indicating no effect on motor behavior. We described the efficacy of DRGS in an earlier publicized study.^36^ In short, DRGS induced a complete reversal of mechanical hypersensitivity during stimulation. A return to pre‐DRGS values was noted after cessation of DRGS at *t* = 60 minutes. Sham‐DRGS did not induce a reversal of mechanical hypersensitivity. Eight out of the 11 DRGS animals responded to DRGS (73%) at *t* = 15 minutes and 10 out of 11 (91%) at *t* = 30 minutes. All 4 DRGS animals that were selected for the GABA‐IR analyses of the current study were responders‐to‐DRGS. At 15 minutes of DRGS, 2/4 animals (50%) responded to DRGS, while at 30 minutes 4/4 animals (100%) responded. At 60 minutes (30 minutes after cessation of DRGS), 1/4 animals (25%) still displayed a slight effect of the DRGS.

### 3.3 Comparison of mean gray values L4‐L6

**Figure 1:**
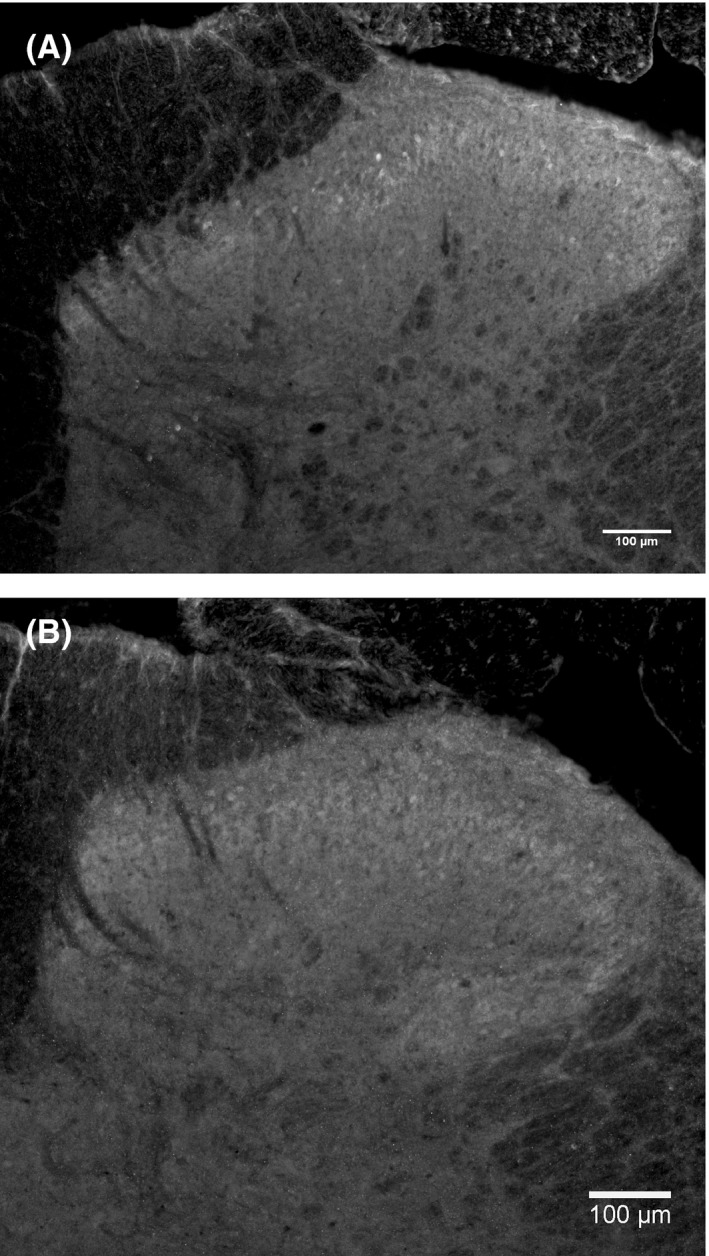
Representative images of the immunohistochemical staining of the upper laminae of the DH in animals implanted with DRGS electrode. No differences in GABA‐IR were observed for the Sham‐DRGS group (A) and DRGS group (B) in the upper laminae of the L4‐L6 spinal segments. Additionally, no differences in GABA‐IR were observed between the ipsilateral DH in both the Sham‐DRGS and DRGS group. Scale bar = 100 μm. GABA, γ‐aminobutyric acid; DRGS, dorsal root ganglion stimulation; DH, dorsal horn; GABA‐IR, GABA‐immunoreactivity

**Figure 2:**
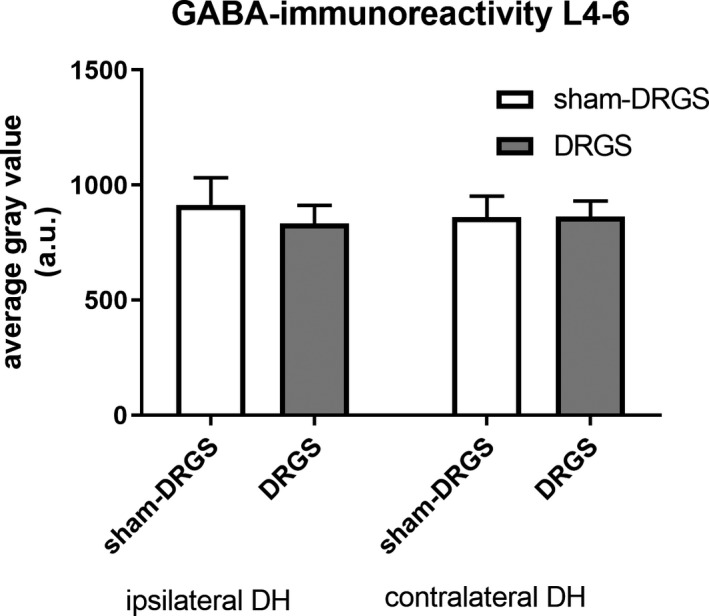
Average gray values of both the ipsilateral and contralateral dorsal horn in lamina 1‐3 of spinal level L4‐L6. Data are expressed as means ± SEM. GABA, γ‐aminobutyric acid; DRGS, dorsal root ganglion stimulation; DH, dorsal horn

The anti‐GABA immunohistochemical analysis showed a strong GABA‐IR, prevalently in laminae 1‐3 of the spinal dorsal horn (Figure 1, the contralateral staining results are not shown). No differences were found between ipsilateral and contralateral GABA‐IR for both the DRGS group and the Sham‐DRGS group in lamina 1‐3 on spinal level L4‐L6 (Figure 2). Additionally, no differences in GABA‐IR were found between DRGS and Sham‐DRGS in lamina 1‐3 of spinal level L4‐6, neither ipsilaterally nor contralaterally (Figure 2).

### 3.4 Comparison of mean gray values per level

**Figure 3:**
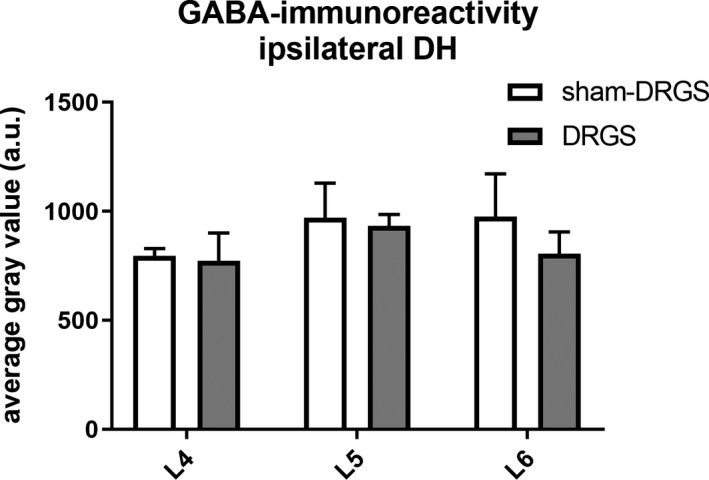
Average gray values of the ipsilateral dorsal horn in lamina 1‐3 per spinal level (L4, L5 and L6). Data are expressed as means ± SEM. GABA, γ‐aminobutyric acid; DH, dorsal horn; DRGS, dorsal root ganglion stimulation

No differences in terms of ipsilateral GABA‐IR were observed between DRGS and Sham‐DRGS on all analyzed levels (L4: *P* > 0.99; L5: *P* > 0.99, L6: *P* = 0.77). Similarly, no differences were found in terms of ipsilateral GABA‐IR for both DRGS (L4 vs. L5 *P* = 0.64; L4 vs. L6 *P* = 0.98; L5 vs. L6 *P* = 0.78) and Sham‐DRGS (L4 vs. L5 *P* = 0.60; L4 vs. L6 *P* = 0.58; L5 vs. L6 *P* > 0.99) between each analyzed level (Figure 3).

**Figure 4:**
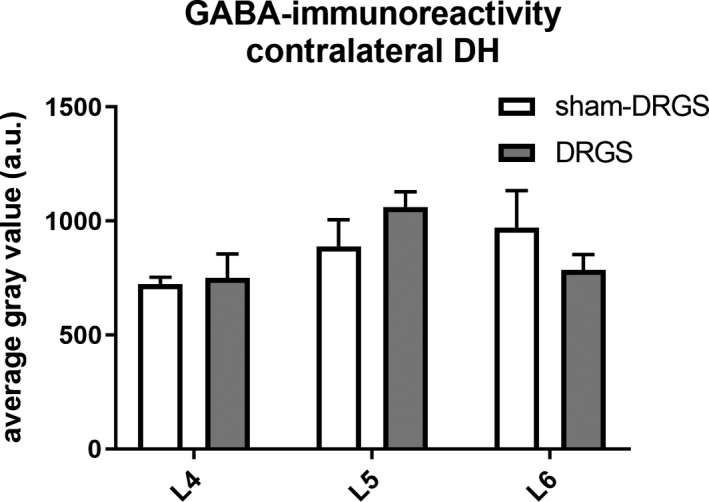
Average gray values of the contralateral dorsal horn in lamina 1‐3 per spinal level (L4, L5 and L6). Data are expressed as means ± SEM. GABA, γ‐aminobutyric acid; DH, dorsal horn; DRGS, dorsal root ganglion stimulation

No differences in terms of contralateral GABA‐IR were observed between DRGS and Sham‐DRGS on all analyzed levels (L4: *P* > 0.99; L5: *P* = 0.57, L6: *P* = 0.58). Similarly, no differences were found in terms of contralateral GABA‐IR for both DRGS (L4 vs. L5 *P* = 0.13; L4 vs. L6 *P* = 0.99; L5 vs. L6 *P* = 0.25) and Sham‐DRGS (L4 vs. L5 *P* = 0.60; L4 vs. L6 *P* = 0.27; L5 vs. L6 *P* > 0.92) between each analyzed level (Figure 4).

## 4 DISCUSSION

This is the first experimental study to assess intracellular GABA levels in the spinal dorsal horn at the peak of the DRGS pain relieving effect in a neuropathic pain model of PDPN. The results of this study indicate that DRGS does not result in decreased levels of intracellular GABA‐IR in the spinal dorsal horn and thus does not induce GABA release. Dorsal column SCS has been demonstrated to induce GABA release from the spinal dorsal horn cells in agreement with the Gate Control Theory,^18^, ^27^ while our results instead point to a different mechanism involved in DRGS and its production of pain relief. The assay and data analysis utilized during our study were performed in an identical way as the previous study regarding GABA release in SCS,^27^ with the difference that the previous study did not concern PDPN animals but neuropathic pain animals with a partial sciatic nerve ligation (according to the Seltzer model). From these data, it was concluded that the assay used for this study is sensitive enough to detect changes in GABA‐IR in the spinal dorsal horn.

The dorsal horn is an essential and second‐order relay station for the integration and modulation of pain.^16^ The Gate Control Theory includes the pivotal role of inhibitory GABA‐ergic interneurons modulating the nociceptive afferents and with this acts as the major regulatory component of the mechanism underlying SCS of dorsal columns.^16^, ^18^, ^19^, ^20^, ^27^, ^38^ Electrical stimulation of the ascending branch of the non‐nociceptive large Aβ fibers located in the dorsal column induces antidromic activation and generates synaptic interactions with GABA‐ergic interneurons that inhibit the transmission of nociceptive signals entering the spinal dorsal horn via the slow‐conducting C fibers.^16^, ^38^ The GABA‐ergic inhibitory interneurons in the superficial laminae of the dorsal horn can be activated by Aβ fiber inputs, which results in enhanced GABA release in the dorsal horn and an increase in extracellular GABA Janssen et al.^27^ Peripheral nerve injury is known to induce a dysfunction of the natural GABA‐ergic inhibition and a neuronal hyperexcitability in the spinal dorsal horn, which are among the major underlying causes of neuropathic pain.^18^, ^30^, ^39^, ^40^, ^41^ The theory that nerve injury induces a loss in GABA‐ergic inhibition in the spinal dorsal horn, causing neuropathic pain, is sustained by experimental studies that showed that pharmacological antagonism of GABA‐ergic inhibition either via the GABA_A,_ but mainly via the GABA_B_ receptor in the spinal cord‐induced mechanical hypersensivity.^42^, ^43^, ^44^ Prior experimental studies confirm that SCS reduces the neuronal excitability and spinal pain transmission.^16^ The role of GABA was furthermore substantiated by experimental and clinical studies demonstrating that the intrathecal application of GABA_B_ receptor agonists like baclofen further potentiate the analgesic effects of SCS,^24^, ^25^, ^26^ while at the same time local perfusion of GABA_B_ receptor antagonists, like bicuculline, abolish this effect.^21^ Therefore, analgesic effects of SCS may be especially attributed to the activation of the GABA_B_ receptor.^21^ An experimental study of Janssen et al^27^ demonstrated additionally a decrease in intracellular dorsal horn GABA‐IR in responders‐to‐SCS, confirming a relation between the release of intracellular accumulated GABA and the analgesic effect of SCS.

Conventional SCS is not successful in all patients, and pain relieving effects can decline over the years.^9^, ^11^ Lack of anatomic specificity of the painful area and positional variations in stimulation is furthermore well‐known drawbacks of conventional SCS therapy. The DRG appears to be an appealing site for neurostimulation,^45^ and clinical evidence indicates that DRGS provides efficacious pain relief in neuropathic pain sufferers,^46^, ^47^, ^48^, ^49^, ^50^ which is confirmed by experimental studies.^35^, ^36^ In comparison with SCS, DRGS has been demonstrated to have a better anatomic specificity of the painful area.^50^ Furthermore, with DRGS the electrical fields have a direct effect on the neural tissues that are pathophysiologically involved in the chronic pain disease condition. Since the location of stimulation is completely different with DRGS (local stimulation at the DRG) as compared to SCS (stimulation of the dorsal column), the question remains whether both stimulation paradigms act via the same mechanism. Our quantitative immunohistochemical data indicate that, even though DRGS and SCS resulted in a similar decrease in PDPN,^36^ DRGS does not act via the stimulation‐induced GABA‐mediated mechanisms in the dorsal horn.

Clearly, DRGS does probably not only directly act via modulation of Aβ fibers, but also of Aδ‐ and C‐type fibers.^15^ As Aβ fibers are stimulated, one might expect that the analgesic effect of DRGS, like that of SCS,^27^ is also linked to activation of intracellular GABA release in the dorsal horn. However, the results of the current study suggest that the analgesic effect of DRGS is not linked to GABA release in the dorsal horn. A hypothetical explanation for this could be that the modulation of a GABA‐mediated Gate Control mechanism with DRGS actually takes place in a primary Gate for nociceptive control, namely at the DRG itself, instead of in the dorsal horn. This hypothesis is supported by a recently published experimental study of Du at al.,^51^ which confirmed that key components of the GABA‐ergic transmission are expressed in the DRG. Their study showed that depolarizing stimuli induce GABA release in the DRG. Additionally, their study demonstrated a reduction in the neuronal excitability in the DRG in response to GABA. Furthermore, focal infusion of GABA or GABA reuptake inhibitors into the DRG alleviated neuropathic pain, and the delivery of GABA_A_ receptor antagonists to the DRG on the other hand exacerbated peripherally induced nociception.^51^ These results indicate that there is a endogenous GABA‐ergic control in the DRG,^52^, ^53^ and analgesic effects of focally applied GABA mimetics suggest that DRGS acts via the modulation of a GABA‐mediated Gate Control at the level of the DRG.

In conclusion, DRGS does not induce GABA release in spinal dorsal horn of neuropathic (PDPN) rats. With this observation, we suggest that the mechanism underlying DRGS‐induced pain relief is different from that of dorsal column SCS. Further research is warranted to elucidate the mechanism underlying DRGS in pain relief. The modulation of a GABA‐mediated “Gate Control” in the DRG, functioning as a prime Gate of nociception, is suggested.

## DISCLOSURE

This work was financially supported by a research grant from Abbott (to P Maino and EA Joosten). The funders had no role in study design, data collection and analysis, decision to publish, or preparation of the manuscript. B Linderoth serves as a consultant to Medtronic; St. Jude Medical/Abbott; Boston Scientific, and Elekta AB. EA Joosten serves as a consultant for Boston Scientific, Medtronic, and Salvia BioElectronics.
